# U-vein compressor improves early haemodynamic outcomes in radiocephalic arterio-venous fistulae in under 2-mm superficial veins

**DOI:** 10.5830/CVJA-2015-008

**Published:** 2015

**Authors:** Mustafa Seren, Omer Faruk Cicek, Mustafa Cuneyt Cicek, Ali Umit Yener, Mahmut Ulaş, Muharrem Tola, Alper Uzun

**Affiliations:** Department of Cardiovascular Surgery, Diskapi Yildirim Beyazit Education and Research Hospital, Ankara, Turkey; Department of Cardiovascular Surgery, Turkiye Yuksek Ihtisas Education and Research Hospital, Ankara, Turkey; Department of Cardiovascular Surgery, Turkiye Yuksek Ihtisas Education and Research Hospital, Ankara, Turkey; Department of Cardiovascular Surgery, Turkiye Yuksek Ihtisas Education and Research Hospital, Ankara, Turkey; Department of Cardiovascular Surgery, Turkiye Yuksek Ihtisas Education and Research Hospital, Ankara, Turkey; Department of Radiology, Turkiye Yuksek Ihtisas Education and Research Hospital, Ankara, Turkey; Department of Cardiovascular Surgery, Ankara Education and Research Hospital, Ankara, Turkey

**Keywords:** arterio-venous fistula, vein diameter, flow, maturation

## Abstract

**Aim:**

In this study, we sought to determine the early postoperative results of arterio-venous fistulae (AVF) created by U-vein compressors with veins between 1.5 and 2 mm in size.

**Methods:**

Pre-operative venous mapping was done. The fistula tract was marked at 0-, 4-, 8- and 12-cm points; 0 cm was the estimated point where the anastomosis would be done. With Doppler ultrasonography, transverse diameters in the estimated fistula tract were measured at the 0-, 4-, 8- and 12-cm points. A superficial vein that would be used as the fistula tract was dilated using U-vein compressors. In the first postoperative hour, the flow in the anastomosis, and the transverse diameter of the fistula tract at the 0-, 4-, 8- and 12-cm points were measured by Doppler ultrasonography.

**Results:**

Forty patients were included in the study. U-vein compressors were used for 20 patients. Postoperative expansion of vein diameters and postoperative flow velocities were found to be statistically significantly different in patients where a U-vein compressor had been used (*p* < 0.001).

**Conclusion:**

We present a technique to dilate veins that are between 1.5 and 2 mm in diameter, which are generally accepted as poor vessels to create radiocephalic arteriovenous fistulae.

## Abstract

The radiocephalic arterio-venous fistula (RCAVF) has remained the access point for maintenance haemodialysis because of its low incidence of complications and high long-term patency rate. Distal radial-cephalic anastomosis just above the wrist is still the best site for an arterio-venous fistula (AVF). This provides a relatively long, straight cephalic vein for catheter insertion. It also leaves more proximal sites for future use should the radialcephalic fistula fail.

A ‘failure-to-mature’ AVF is caused by intrinsically poor native vessels. Poor native vessels relate to the use of a suboptimal artery or vein to create the AVF.^1^

## Methods

Between January 2010 and April 2012, 40 normotensive patients (mean age 56.8 years, range 47–69), 22 males and 18 females, who underwent RCAVF (20 patients by standard technique, 20 by modified technique) were included in this study. The following inclusion criteria were considered before access placement: (1) the non-dominant arm should be selected (if possible), (2) the access should be placed distally in the forearm, (3) the selected veins in the forearm should have a long segment to allow for variation in puncture sites and should have a diameter between 1.5 and 2 mm. Exclusion criteria were: (1) atherosclerotic or calcific arteries, (2) redo operations, (3) hypotensive patients.

**Fig. 1.**
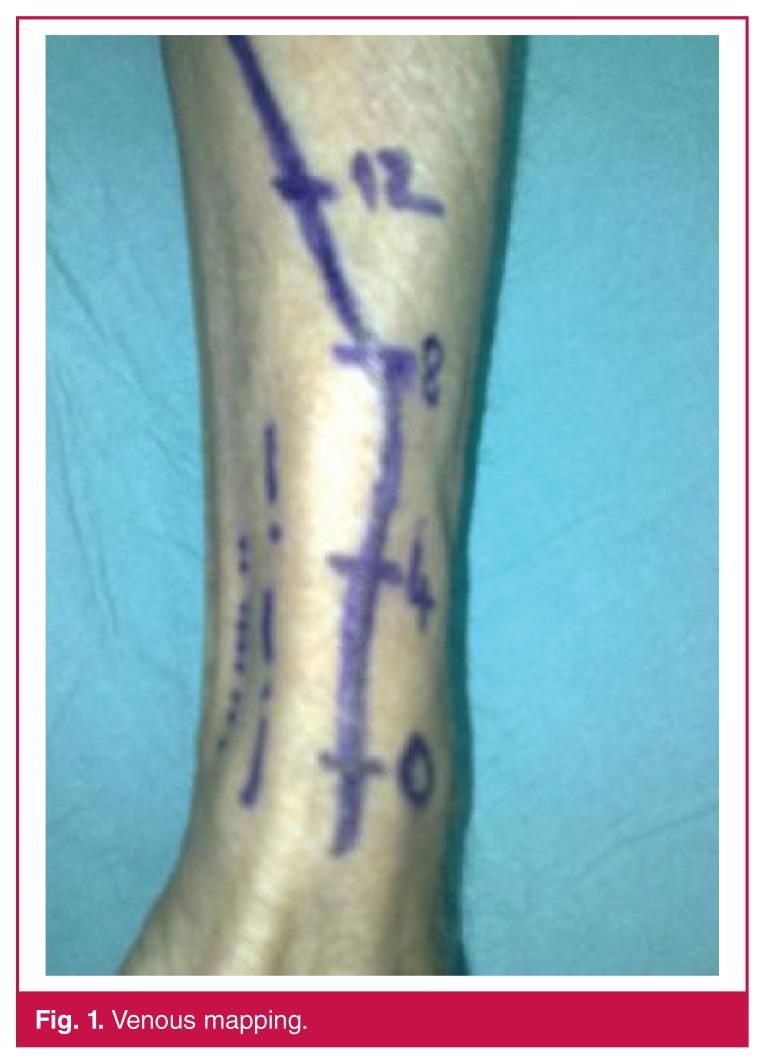
Venous mapping.

Pre-operatively, venous mapping was done on all patients. The fistula tract was marked at 0-, 4-, 8- and 12-cm points (Fig. 1)); 0 cm was the estimated point where the anastomosis would be done. With Doppler ultrasonography, the arterial and venous systems were examined and transverse diameters were measured at the 0-, 4-, 8- and 12-cm points.

All operations were done under local anaesthesia. The cephalic vein was dissected surgically and freed in the distal forearm. Then distal end was ligated with a silk suture. An intravenous catheter was introduced through the proximal end of the vein and 2 500 IU of diluted heparin was transfused into the vein. For the standard technique (20 patients), a 10–12-mm arteriotomy was done in the radial artery and the cephalic vein was anastomosed end to side to the radial artery.

**Fig. 2.**
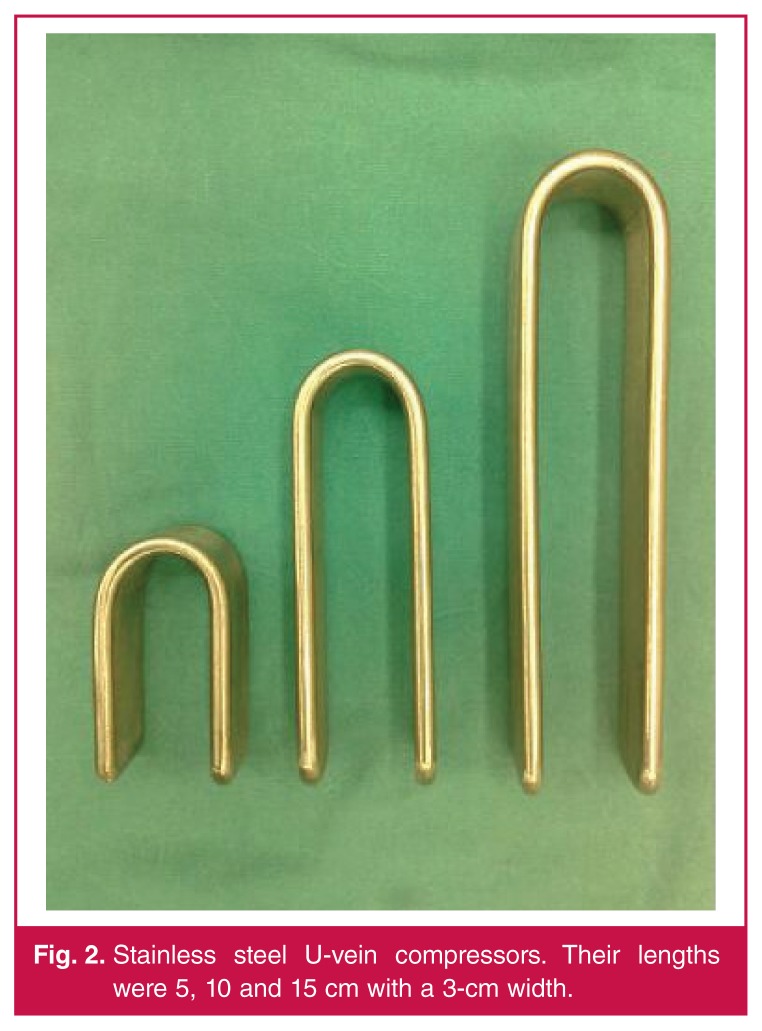
Stainless steel U-vein compressors. Their lengths were 5, 10 and 15 cm with a 3-cm width.

**Fig. 3.**
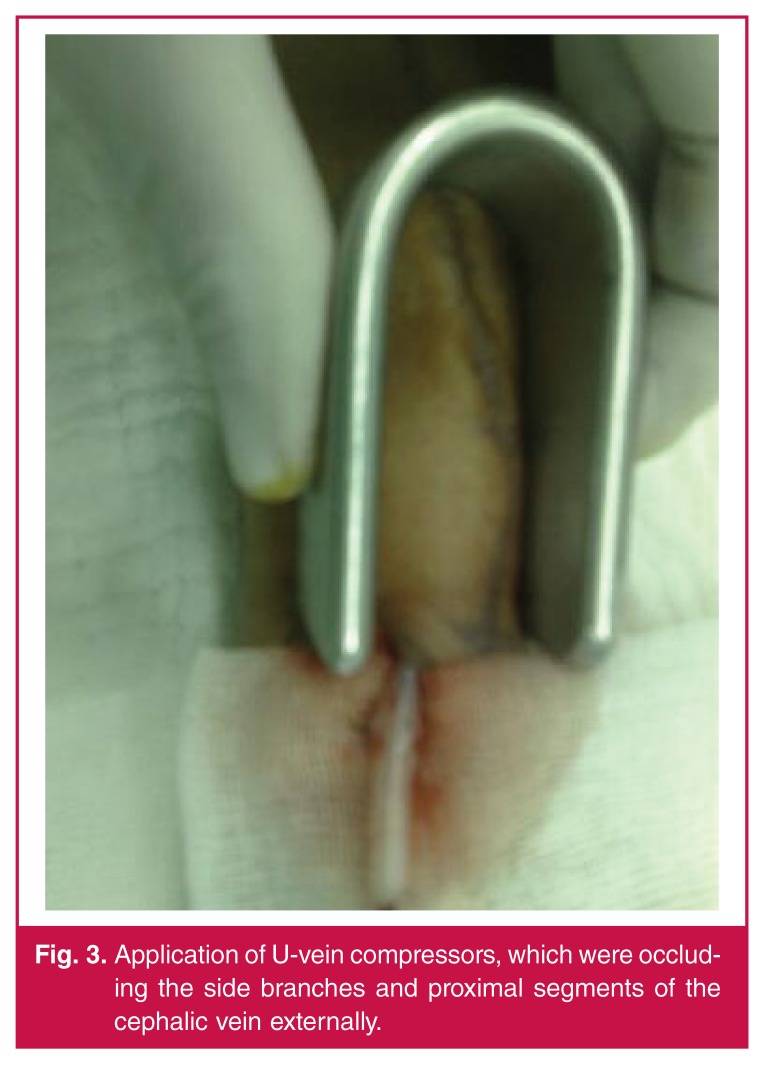
Application of U-vein compressors, which were occluding the side branches and proximal segments of the cephalic vein externally.

In the modified technique (20 patients), we used U-vein compressors manufactured from stainless steel, 3 cm in width and 5, 10 and 15 cm in length (Fig. 2). Here, a superficial vein that would be used as the fistula tract was dilated with the U-vein compressors by injecting a saline solution just after the intravenous catheter was introduced through the proximal end of the vein (Fig. 3). All sizes of U-vein compressors were used successively to dilate the vein gradually. The U-vein compressors occluded side branches and proximal segments of the cephalic vein externally. A 10–12-mm arteriotomy was placed in the radial artery and the cephalic vein was anastomosed end to side to the radial artery.

Postoperatively, the presence of a trill in the arteriovenous fistula was examined by palpation. In the first postoperative hour, flow in the anastomosis and the transverse diameter of the fistula tract at the 0-, 4-, 8- and 12-cm points were measured with Doppler ultrasonography.

## Statistical analysis

Statistical analyses were performed with SPSS 15.0 software (SPSS Inc, Chicago, IL). Forty patients were included in the analysis. Descriptive statistics are presented for numerical variables (mean, standard deviation, median, minimum and maximum). If the comparison of two independent groups of continuous variables provided the assumption of normality, the *t*-test was used. If it did not provide the assumption of normality, the Mann–Whitney *U*-test was used. The repeatedmeasures ANOVA test was used for repeated-measures statistics. All values of *p* < 0.05 were taken as significant.

## Results

Forty patients were included in the study. Comparison of baseline clinical characteristics and pre-operative vein diameters between the two groups are shown in Table 1. U-vein compressors were used in one group of 20 patients (10 males, mean age 57.9 ± 8.12 years) and not in the other group of 20 patients (12 males, mean age 55.8 ± 7.52 years). There were no significant differences in age, gender, history of hypertension, hyperlipidaemia, diabetes mellitus, chronic obstructive pulmonary disease, coronary artery disease, current smoking status and pre-operative vein diameters between patients who were operated with and without the U-vein compressor.

**Table 1:** Comparison of patients where U-vein compressors were not used and those where they were used

*Variable*	*Patients (U-vein compressor not used) (n = 20)*	*Patients (U-vein compressor used) (n = 20)*	p-value*
Age (years)	55.8 ± 7.52	57.9 ± 8.12	0.41
Male, *n* (%)	12 (60)	10 (50)	0.53
Hypertension, *n* (%)	7 (35)	8 (40)	0.74
Hyperlipidaemia, *n* (%)	9 (45)	7 (35)	0.52
Chronic obstructive pulmonary disease, *n* (%)	5 (25)	6 (30)	0.72
Coronary artery disease, *n* (%)	7 (35)	6 (30)	0.74
Diabetes mellitus, *n* (%)	5 (25)	6 (30)	0.72
Current smoker, *n* (%)	5 (25)	6 (30)	0.72
Pre-operative vein diameter	1.77 ± 0.11	1.79 ± 0.12	0.71

There were no significant differences in the pre-operative diameters of the veins at the 0-, 4-, 8- and 12-cm points in the groups. Postoperative vein diameters in the patients where U-vein compressors were used were significantly greater at all points, compared with patients where it was not used (*p* < 0.001). Also, postoperative flow velocities were significantly higher in patients where U-vein compressors were used (*p* < 0.001) (Table 2).

**Table 2:** Comparison of pre- and postoperative diameters and postoperative flow velocity between patients where the U-vein compressor was not used and those where it was used

	*Patients (U-vein compressor not used) (n = 20)*	*Patients (U-vein compressor used) (n = 20)*	*p-value*
0 cm pre-operative	1.77 ± 0.11	1.79 ± 0.12	0.709
0 cm postoperative	2.45 ± 0.25	3.27 ± 0.42	< 0.001
4 cm pre-operative	1.78 ± 0.11	1.79 ± 0.11	0.832
4 cm postoperative	2.45 ± 0.25	3.27 ± 0.40	< 0.001
8 cm pre-operative	1.78 ± 0.11	1.81 ± 0.10	0.282
8 cm postoperative	2.47 ± 0.26	3.33 ± 0.38	< 0.001
12 cm pre-operative	1.79 ± 0.11	1.83 ± 0.10	0.307
12 cm postoperative	2.46 ± 0.26	3.33 ± 0.36	< 0.001
Flow velocity (postoperative)	197.15 ± 53.52	371.75 ± 93.98	< 0.001

In patients where the U-vein compressor was used, pre-operative mean transverse diameters (range 1.60–1.95 mm) at the 0-, 4-, 8- and 12-cm points were increased at least 75% postoperatively (range 2.80–3.90 mm). Flow measurements were between 326 and 670 ml/min.

## Discussion

Endogenous AVF, first described in 1966, remains the optimal vascular access for chronic dialysis.^2^ The RCAVF has remained the access for maintenance haemodialysis because of its low incidence of complications and high long-term patency rate.

An AVF within the anatomical snuffbox (triangular deepening on the radial, dorsal aspect of the hand) has a high incidence of early failure and requires a longer maturation time. The proximal elbow fistula predisposes to ischaemic complications and can lead to congestive heart failure as a result of increasing flow through a chronic fistula that is made too large. The distal radial–cephalic anastomosis just above the wrist is still the best site for an internal AVF. This provides a relatively long, straight cephalic vein for catheter insertion. It also leaves more proximal sites for future use should the RCAVF fail.

The fistula is allowed to mature for six to eight weeks prior to puncture. Occasionally longer periods of maturation are required to allow sufficient arterialisation of the vein, but if little venous distention is present at six weeks, either revision or an alternate access site is usually required. Having the patient perform repetative hand exercises such as squeezing a ball or a similar-sized compressable object may facilitate development of the outflow vein.

A failure-to-mature AVF is caused by intrinsically poor native vessels or by post-surgical derangements. Poor native vessels relate to the use of a suboptimal artery or vein to create the AVF. It has been noted that arteries less than 1.5 to 2 mm and veins less than 2 to 2.5 mm in diameter are associated with poor AVF maturation.^3^-^6^ Silva *et al*. used a minimum of 2.5-mm vein size as predictable for fistula success.^1^ In our technique, veins between 1.5 and 2 mm were associated with good AVF maturation by intra-operative use of a U-vein compressor.

Larger veins mean larger flow. However, such a simplistic view does not take into account arterial factors and normal pulsatile blood flow. Furthermore, venous compliance after fistula creation needs to be considered. In the study by Lauvao *et al.*, eight patients with the smallest diameter between 1.5 and 2 mm on Doppler ultrasonograhy went on to develop mature fistulae, and three did not.^7^ Their experience shows that vein size is the major predictor for a succesfull fistula.

In our study, pre-operative mean transverse diameters (range 1.60–1.95 mm) at the 0-, 4-, 8- and 12-cm points were increased at least 75% postoperatively (range 2.80–3.90 mm) and flow measurements were between 326 and 670 ml/min. The risk of failure was zero in the group where U-vein compressors were used, but the wrist radiocephalic arterio-venous fistula failed in six in the other group.

A well-functioning vascular access for haemodialysis plays a key role in the quality of life and clinical outcome of dialysis patients. Johnson *et al.* reported that a high intra-operative flow volume defined as 320 ml/min or greater was associated with a lower number of surgical revisions and longer access survival regardless of gender, race and the presence of diabetes.^8^ The same authors reported that an intra-operative flow rate of less than 170 ml/min was correlated with a 56% risk for AVF failure within 50 days of construction.^8^ A recent study including a cohort of 109 patients undergoing vascular access surgery for first-time haemodialysis showed that an intra-operative flow rate greater than 200 ml/min was associated with better mid-term outcomes in terms of requirement for revision and early patency rate.^9^

Fistula maturation is defined by the determination of both vascular surgeon and nephrologist that an access is patent and ready for cannulation based on adequency of blood flow through the fistula and adequency of vein dilatation in the 10-cm segment.^7^ In our technique, U-vein compressors were occluding side branches and proximal segments of the cephalic vein externally. The cephalic vein was dilated in the approximately 10–12-cm segment and its compliance was increased.

In most succesful AVFs, these flow and size parameters are generally met within the first few weeks of construction.^10^ In our technique, these parameters (diameter and flow) are met in the first few minutes postoperatively because the superficial fistula tract is dilated enough during the operation by intra-operative use of the U-vein compressor.

We believe that this technique can maximise flow and minimise failure in AVFs using small, superficial veins. Postoperative hand exercises were not needed to accelerate maturation. This study needs long-term follow up but the technique we have described could be an alternative in patients with poor venous networks in the forearm.

## Conclusion

In this article, we present a technique to dilate veins of between 1.5 and 2 mm in diameter, which are normally accepted as poor vessels to create RCAVF. With this technique, we can create good functioning arterio-venous fistulae in the early postoperative period, even if the superficial veins are not suitable for the standard technique. Our preliminary experience has shown satisfactory outcomes compared to the standard technique.
